# Improved Exopolymer Production by *Chromohalobacter canadensis* Cultures for Its Potential Cosmeceutical Applications

**DOI:** 10.3390/microorganisms8121935

**Published:** 2020-12-06

**Authors:** Nadja Radchenkova, Merve Erginer Hasköylü, Spasen Vassilev, Songül Yaşar Yıldız, Ivanka Boyadzhieva, Ebru Toksoy Oner, Margarita Kambourova

**Affiliations:** 1 The Stephan Angeloff Institute of Microbiology, Bulgarian Academy of Sciences, Acad. G. Bonchev Str. Bl. 26, 1113 Sofia, Bulgaria; nstoicheva@yahoo.com (N.R.); spasen.vasilev@abv.bg (S.V.); petrovaim@abv.bg (I.B.); 2 IBSB, Department of Bioengineering, Faculty of Engineering, Marmara University, Goztepe, Istanbul 34722, Turkey; merweerginer@hotmail.com (M.E.H.); ebru.toksoy@marmara.edu.tr (E.T.O.); 3 Department of Bioengineering, Faculty of Engineering and Natural Sciences, Istanbul Medeniyet University, Kadıköy, Istanbul 34720, Turkey; songulyasar@ymail.com

**Keywords:** halophilic bacterium, exopolymer, continuous cultivation, dilution rate, biocompatibility

## Abstract

Several exopolymers with different chemical composition and correspondingly variety in their physico-chemical properties from halophilic microorganisms have still been described, however, with a low production yield. *Chromohalobacter canadensis* 28 isolated from Pomorie saltern synthesized an unusual exopolymer (EP) containing 72% γ-polyglutamic acid (PGA), an essential cosmeceutical additive. Current work suggests a novel approach for effective EP synthesis by *C. canadensis* 28 using continuous cultures. Highest production was observed at low dilution rates reaching a level of 2.1 mg/mL at D = 0.035, similar to those in batch cultures (2.34 mg/mL), however avoiding all disadvantages of discontinuous fermentation processes. At steady state, the total quantities of the synthesized EP after 48 h cultivation for the given equipment volume in D = 0.035 h^−1^ and D = 0.075 h^−1^ were 8.67 and 12 g, correspondingly, while it was 2.9 g for batch culture. Process parameters did not change after a ten-day run at D = 0.35 h^−1^. A degree of purity of EP fraction received from continuous cultures was significantly increased up to 93–96%. A lack of cytotoxicity and high cell viability were observed for human dermal fibroblast cells after 24 h incubation with crude EP from *C. canadensis* 28 and purified PGA fraction that could suggest its high potential for cosmetic applications.

## 1. Introduction

Halophilic bacteria have developed various strategies to avoid water loss at high salt concentration, and they possess unique physiological mechanisms for adaptation like production of bacteriorhodopsin, halorhodopsin, compatible solutes, polyhydroxyalkanoates (PHA), enzymes, or ether-linked lipids [1,2].

Exopolymer (EP) synthesis is one of the important features of many halophilic microbes to survive in a saline environment. Microbial polymers express a variety in their chemical composition and correspondingly their physico-chemical properties. The main disadvantages associated with microbial fermentation processes include preliminary vessel sterilization, the providing of air during the process, the maintaining of a constant bioprocess temperature, and expensive media components that result in high production costs [3,4]. Additionally, microbial processing is characterized by low biomass and respectively low EP synthesis in comparison with synthetic and plant-based polymers [5]. Halophilic producers suggest many process advantages as their products are not pathogenic; their rigid molecules remain their properties at extreme salinity like many industrial processes are; and high salinity in the medium permits open processes without contamination risks [2,6]. Although a number of new molecules with interesting properties have been isolated from halophiles, their biotechnological prosperity is still strongly neglected [7].

Difficulties encountered in halophilic cultivations and lower production yield determine the difficult method of a laboratory-scale for the actual development of pilot and large-scale production. Discontinuous fermentation processes are preferable for maintaining the sterile process; however, they are not economically effective enough [2]. Exploration of continuous cultures provides constant productivity of the system by maintaining the microbial producers inside while cell products pass through the system with constant product quality and quantity accompanied by lower accumulation of wastes or bioproducts. The advantage of this system is that the microbial population within the vessel grows at a constant rate in a constant environment and assumes a “steady state” in long-term experiments. Continuous bioprocessing suggests a decrease in energy consumption, low labor cost, and effective reactor utilization by elimination of the time for cleaning and sterilization of the vessel and the comparatively long lag phases before the organisms enter a brief period of high productivity. While the continuous culture technique has a long history, its exploration in cultivation of halophilic microorganisms is quite limited and refers to production predominantly of PHAs, including PHB [3,8,9]. To the best of our knowledge, the bioprocess of continuous production of exopolysaccharides or polypeptides by halophilic bacteria has not yet been described.

*Chromohalobacter canadensis* 28 isolated from Pomorie saltern was described in our previous work as a producer of an unusual exopolymer (EP) containing both EPS and γ-polyglutamic acid (PGA) fractions [10]. The prosperity of continuous cultivation for effective microbial production and lack of such information for EPSs synthesized by halophilic microorganisms sparked our interest in the development of a novel approach for EP synthesis by *C. canadensis* 28 using continuous cultures.

By this strategy, we attempted to determine kinetic parameters of growth and EP production, besides improving the polymer production.

## 2. Materials and Methods

### 2.1. Bacterial Strain

*C. canadensis* 28 (NBIMCC 8836) was isolated from Pomorie salterns (42°35′33″ N, 27°37′21″ E), Black Sea, Bulgaria and deposited in NBIMCC (WDCM NO 135) under the number 8924 [10]. The strain was grown in an optimal medium consisting of (%): lactose, 1.0; NaCl, 15; tryptone, 1.0; yeast extract, 0.5, pH 7.5. A single colony was transferred in 20 mL of the above media and cultivation was performed on a rotary shaker for 72 h at 30 °C. Then, 3 mL of the culture liquid was transferred into 100 mL fresh medium and incubated for 18 h up to optical density (OD_660_) of 2–2.5. The bioreactor containing the same medium was inoculated with 3.0% (*v/v*) seed culture under aseptic conditions.

### 2.2. Operation Conditions

Fermentations were conducted in a 5 L jacketed glass reactor (LF-2, Prague, Czech Republic) equipped with a hydrofoil impeller Narcissus (0.05 m diameter) filled with 2.4 L medium at 900 rpm and 1.0 vvm, both determined as the optimal agitation speed and aeration rate, respectively [11]. Dissolved oxygen (DO, %) was provided by injecting filtered air at a flow rate of 2.4 L/min and followed in all runs by InPro 6800/12/320 Mettler Toledo Ingold (Leicester, LE4 1AW, UK) oxygen sensor. The temperature was maintained by a regulation system at 55 °C throughout the experiment. pH value was observed to increase from 7.2 after sterilization to 7.8 during the exponential phase and was constant during the process of about 7.4. Foaming was controlled by addition of Antifoam 204 (Sigma-Aldrich, Taufkirchen, Germany). Batch fermentation was performed up to 3.7 (OD_660_) before the start of continuous operation. The value for dilution rate (D) was established by the rate of flow (F) of nutrient medium through the chemostat of constant volume (V), D = F/V. The feed medium was transferred to the fermentation vessel and the volume was maintained constant (2.4 L) by peristaltic pumps. Sampling was done at an interval of 3 h during batch stage and of 6 h during continuous cultivation. Continuous cultivation was performed at five different dilution rates (D), 0.02, 0.035, 0.05, 0.075, and 0.1 h^−1^. Dilution rates providing highest production (D 0.035) and highest productivity (D 0.075) were repeated three times and the rest of the dilution rates were repeated twice.

### 2.3. Analytical Methods

Culture growth was determined by measuring samples’ turbidity at 660 nm. After centrifugation at 4000× *g* for 10 min the pellet was washed with distilled water twice, and then dried to a constant weight. One-unit OD was established to correspond to 1.05–1.1 mg/mL dry cells in all used dilution rates. For EP extraction, the supernatant was treated with an equal volume of cold absolute ethanol added dropwise under stirring in an ice bath, held at −18 °C overnight and then centrifuged at 13,000× *g* for 30 min to separate the precipitate and supernatant. The precipitate was then washed twice with 50% ethanol and redissolved in distilled hot water. Water-insoluble materials were removed by centrifugation, and the sample was dialyzed against distilled water and dried. As EP was established to consist of two fractions, namely, EPS (14.3% *w/w*) and protein (72% *w/w*), all samples were tested for carbohydrate, protein, and nucleic acid contents. The carbohydrate content was determined using the phenol/sulfuric acid method with glucose as a standard [12]. The protein fraction consisting almost entirely of polyglutamic acid [10] was determined by the Lowry test using bovine serum albumin as a standard. EP content was estimated as a sum from EPS and protein content. The degree of purity was estimated as a ratio between the counted EP and dry weight of the samples. Nucleic acid content was calculated by reading UV absorption of samples at 260 and 280 nm. As the established values for nucleic acids content did not exceed 0.9%, they were neglected. The level of residual lactose was measured according to the dinitrosalicylic acid (DNS) method [13] by using lactose as a standard. Twenty mL samples were harvested after at least three residence times until the standard deviation of ≤15% for biomass data and ≤5% for EP data were obtained confirming the steady state condition. Five samples were taken each hour after reaching the steady-state and an average value was presented as a result.

### 2.4. In Vitro Bioactivity Test

To estimate bioactivity of produced crude polymer, purified γ-polyglutamic acid (PGA) from *C. canadensis* 28 and hyaluronic acid (Heze Biochemical, Shandong, China) were tested with Human Dermal Fibroblast Cell Line (PCS-201-012) at different concentrations (0, 10, 100, 500, and 1000 µg/mL) for 24 h. Viability of the cells was investigated with WST-1 (4-[3-(4-iodophenyl)-2-(4-nitrophenyl)-2H-5-tetrazolio]-1,3-benzenedisulfonate) (Roche Applied Science, Penzberg, Germany) cell proliferation kit. Briefly, cells at the 70% confluency were trypsinized and seeded on to a 96-well plate at the cell/well density of 1 × 10^4^. After overnight attachment, the medium (DMEM complete with 10% FBS and 1% penicillin-streptomycin) (PAN Biotech, Aidenbach, Germany) was replaced with an experimental medium that contained EP from *Chromohalobacter canadensis* 28 (CcEP), PGA, or HA and incubated for 24 h at 37 °C in humidified air containing 5% CO_2_. After the incubation period, the WST-1 reagent was added onto wells and incubated at 37 °C for two more hours at dark in 5% CO_2_. The absorbance was measured at 405 nm by GloMax Multi + Microplate Multimode Reader (Promega, Madison, Wisconsin, USA). Untreated cells (cells on wells without any sample but DMEM media) were used as a control and considered as 100% viable. All experiments were performed in triplicate, and statistical analysis of in vitro test data was performed via GraphPad Prism 5 with One-Way ANOVA and Tukey tests. Data were presented as a mean of 95% confidence interval (CI). AP-value below 0.05 was considered statistically significant.

## 3. Results

### 3.1. Effect of the Dilution Rate on EP Production

**Figure 1:**
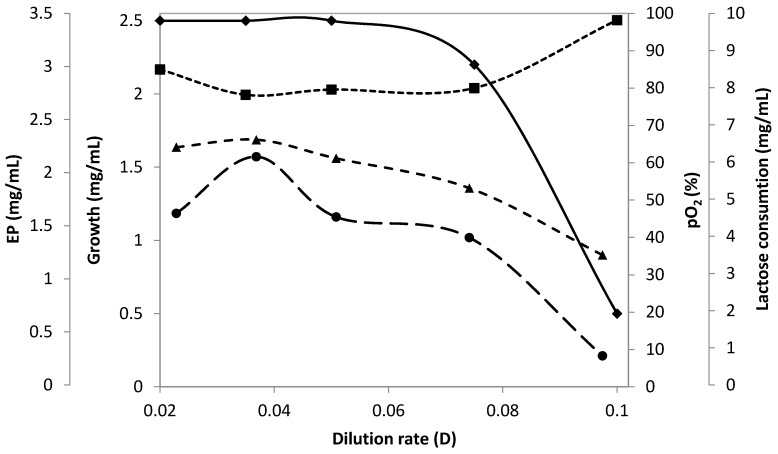
Effect of dilution rate on growth (♦) and exopolymer (EP) production (●) during the continuous cultivation of *C. canadensis* 28 in a stirred-tank fermenter at aeration of 1.0 vvm and agitation rate 900 rpm. Changes in the consumed lactose (▲) and dissolved oxygen (■) were registered.

Following our previous results, cultivation of *C. canadensis* 28 was performed in an optimal medium for growth and EP production 900 rpm and 1.0 vvm [10,11]. The time course of batch fermentation continued up to the 18th hour of cultivation, when OD_660_ of 3.7 (1.7 mg/mL) was measured, pO2 of 75%, then continuous experiments were performed with dilution rate ranging from 0.02 to 0.1 h^−1^. The dry biomass concentration was constant at lower dilution rates (D = 0.02–0.05), 2.5 mg/mL (Figure 1). Increased dilution rates (D = 0.075 h^−1^) caused slight biomass decrease (2.2 mg/mL) and significant decrease at dilution rate of 0.1 h^−1^ (0.5 mg/mL) revealing that growth at this dilution rate was close to the washout condition. EP synthesis was highly influenced by the medium flow speed suggesting partially growth associated production. Among the tested dilution rates, the highest EP production was observed at D = 0.035 h^−1^. At the highest D = 0.1 h^−1^, EP content roughly correlated with the low biomass concentration due to cell washout and moderately increased with a dilution rate decrease reaching a peak of 2.1 mg/mL at D = 0.035 h^−1^, followed up by a decrease at D 0.02 h^−1^. The synthesized EP at D = 0.035 h^−1^ was almost eight-fold higher in comparison to that obtained at D = 0.1 (0.27 mg/mL).

The used lactose (S) for different dilution rates changed in the range 5.3–6.6 mg/mL for D = 0.02–0.075 (initial concentration of 10 mg/mL) (Table 1) while it was 7 mg/mL for batch culture. The highest value of 6.6 was measured for D = 0.035 when a highest EP level was registered. Despite high productivity observed at D = 0.075, the incoming fluid rate of 180 mL/h compensated carbon source utilization, and a level of 5.3 mg/mL lactose was registered. At D = 0.1 h^−1^, the consumed lactose diminished considerably in parallel with low biomass concentration.

As productivity in continuous cultures depends on both EP/biomass ratio but also its dilution rate, it increases with increasing D. The level of residual oxygen was almost constant with the exception of a case with slow metabolism (D = 0.02 h^−1^) and cell washout (D = 0.1 h^−1^) (Figure 1). A continuous process was run at D = 0.35 h^−1^ for ten days without a substantial change in process parameters including productivity.

The values for EP productivity significantly differed for the tested D. It was highest at D = 0.075 h^−1^ and significantly decreased at lowest and highest D (>300%). Maximum productivity was increased by approximately 250% in continuous cultures compared to batch culture.

Together with a drastic increase of continuous cultures productivity in comparison with batch cultures, an increased content of EP in the pellet received after ethanol precipitation (determined as a sum of EPS and protein content) was observed. EP represented 78% of the polymers in the pellet received from batch cultures while this content significantly increased at continuous cultures. A lowest value of 88% was received at D = 0.02 probably as a result of some lysis at a low dilution rate while, in the rest area of D, it was 93–96% (Table 2). Consequently, continuous cultivation facilitates the stage of EP purification resulting in the lowering of the final trade price.

### 3.2. In Vitro Bioactivity Results

**Figure 2:**
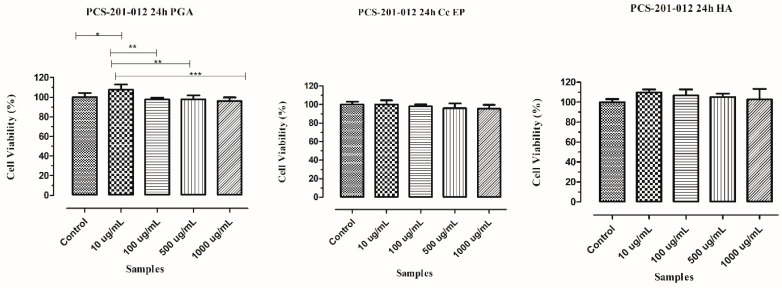
Viability results of pcs-201-012 (Human Dermal Fibroblast Cell line) cells after being cultivated with EP from *C. canadensis* 28, PGA, and HA (AP value below 0.05 is represented as (*; 0.01 to 0.05, **; 0.001 to 0.01, and ***; <0.001).

Cell viability results of human dermal fibroblast cells after 24 h incubation with crude EP from *C. canadensis* 28, PGA purified from EP of *C. canadensis* 28 and HA for 24 h are shared in Figure 2.

As a result of WST-1 cell proliferation analysis, cellular viability at the end of 24 h was 100, 107.6, 97.78, 97.81, and 96.18% for control, 10, 100, 500, and 1000 µg/mL doses for PGA samples, respectively. Viability of cells after being cultivated with *C. canadensis* 28 EP for 24 h were recorded as 100, 100.1, 98.11, 96.04, and 95.37% for control, 10, 100, 500, and 1000 µg/mL. After cultivation with HA, viability of the cells was observed as 100, 109.80, 106.80, 105.20, and 102.80% for control, 10, 100, 500, and 1000 µg/mL. PGA showed higher viability than the control group for the dose of 10 µg/mL and almost similar results with the same dose of HA. EP also showed similar results as the control group at a concentration of 10 µg/mL. All samples showed high cell viability similar to HA, and no cytotoxicity was observed.

## 4. Discussion

Variation in dilution rate did not change the strain growth in the range 0.02–0.05 h^−1^; however, there were significantly changed EP levels and productivity. Similarly, the low effect of the other two bioreactor parameters, agitation speed, and aeration rate on the bacterial growth has been observed while their influence on EP production was significant [11].

Average production yield of marine EPS is around 1 g/L while industry requires a production yield around 10 g/L [14]. The EP yield reached by *C. canadensis* 28 in batch cultures above two mg/mL has placed this strain among the best halophilic producers [11]; however, this level is still not enough for development of an industrial process. Exploration of continuous cultivation could be a way for development of an effective EP production at constant conditions avoiding preliminary stages in process run-up like cleaning and sterilization of each new reactor and different phases in cell development. The performed bioreactor experiments revealed an effective production of EP by *C. canadensis* 28 in continuous cultures at low dilution rates. The EP level in continuous cultures (2.15 mg/mL) is similar to that in batch cultures (2.34 mg/mL) at the same kinetic parameters of the bioreactor [11]. Higher levels in continuous cultures in comparison with batch ones were reported for *Lactobacillus delbrueckii* ssp. *bulgaricus* [15] and *Anoxybacillus pallidus* 418 [16]. In continuous cultures of *C. canadensis*, 28 high EP yields were observed at low dilution rates. The requirement for low dilution rates in conventional chemostat processes with halophiles is determined by their low specific growth rates [17]. The observed high synthesis at comparatively low D = 0.035 h^−1^ was also in good agreement with the observed enhanced EP synthesis in the late exponential phase during batch cultivation [11]. Some authors have explained maximal levels of EPS synthesis in continuous cultures at lower dilution rates by the increased residence time of bacteria supporting utilization of excess carbon source for EPS [18]. In our case, the EP level at D = 0.035 h^−1^ was higher than at D = 0.02 h^−1^, which could be due to some metabolic inhibition. The maintaining of the halophilic bacterium culture in a steady state ensured development of a constant system for high level EP production for at least ten days. The total quantity of the synthesized EP after 48 h batch cultivation for the given equipment volume of 2.4 L was 2.9 g while synthesized EP for 48 h at steady state in D = 0.035 was 8.67 g. This value was 12.00 g at D with the highest productivity (0.075). The economic viability of microbial EP production by microorganisms is impaired by the high price of substrates (commonly sugars as sucrose, lactose, and glucose), a requirement for costly downstream processes including the evaluation of media utilization [19]. The conventional process is accompanied by a continuous supply of a fresh medium balanced by the efflux of the spent medium. Productivity at D = 0.075 h^−1^ was about 140% higher in comparison with that at D = 0.035 h^−1^; however, an estimation of the process effectiveness should also include the quantity of the used medium. It was 84 mL/h at D = 0.035 h-1, while, at D = 0.075 h^−1^, it was twofold higher—180 mL/h. A lower degree of substrate utilization at a higher dilution rate and the need for a significantly higher volume of ethanol for precipitation of the bigger culture supernatant could influence the market price of EP produced by *C. canadensis* 28. An additional economic evaluation could enlighten the cost effectiveness of the process at different rates. Using lactose, a by-product from the dairy industry as a carbon source for EP synthesis, could decrease the trade cost significantly in large-scale processes with *C. canadensis* 28. Unusually high NaCl concentration (15%) could favor less sterility care or even an open process for EP production.

Poly-gamma-glutamic acid (γ-PGA) is a naturally occurring, biodegradable, edible, nontoxic, environmentally friendly, and nonimmunogenic poly-amino acid [20]. Several bacteria like *Bacillus subtilis* (natto), *B. licheniformis*, *B. amyloliquefaciens*, *B. pumilis*, *B. megaterium*, *B. mojavensis*, *B. anthracis* [21], and halophilic archea *Natrialba aegyptiaca* [22] produce this compound. The only reported halophilic bacterial PGA producer has been *C. canadensis* 28 [10]. PGA properties determined our interest in a compound consisting mainly of PGA (72%). Hydrophilic, biocompatible hydrogels of γ-PGA with good swelling behavior and flexibility similar to natural tissues are suitable for various biological applications including drug carrier systems, bioadhesives, cryopreservation, tissue engineering, and cosmetic applications. Its chemical stability is widely preferred in cosmetics for enhancing the novelty of personal care products like moisturizers, winkle-removers, or exfoliants. PGA is known to form a soft, smooth, self-elastic and moisturizing film on skin and stimulate natural moisturizing factors, and its 3D molecule structure network allows for high water retention with humectant activity [10,21,23,24,25,26,27]. In another study, an enhanced human-derived fibroblasts proliferation has been observed after being treated with a skin care sponge, containing poly-(γ-glutamic acid) [28]. PGA esters have been observed to modify collagen gels on human skin fibroblast cell lines HFF-1 and 3T3 [29,30]. Hua et al. [31] have shown 117.6% biocompatibility of pH sensitive PGA (γ-PGA) and ε-polylysine (ε-PL) hydrogels when cultivated with a murine fibroblast cell line (L929). High biocompatibility, cellular proliferation, and migration of L929 cells have been reported after treatment with multilayer films containing PGA [32]. Sun et al. [33] have observed an improved cell viability of infected SD rat skin wound model and NIH 3T3 embryomic mouse fibroblast cells after treatment with PGA esters. Hyaluronic acid (HA) was included as a control in our experiments. Together with PGA, HA is among the most common and very important components for skin care products with its cell proliferation, collagen proliferation, skin augmentation, and moisturizing properties, highly preferred by both producers and consumers. Ben-Zur and Goldman [27] have concluded that γ-PGA as hydrophilic humectant enhances skin elasticity better than collagen and HA. Another component of EP from *C. canadensis* 28, EPS fraction (14.3%) is also an extremely important skin active ingredient, which provides moisturizing, whitening, instant tightening, restorative, anti-age, and protective effects on the skin. The origin of the biopolymer from extremophilic microorganisms determined its safety for human health, its effective skin action at different temperatures, and its long-lasting endurance. In our previous study [11], high viability of another cell line, the human keratinocyte cell line (HaCaT), has been observed after their treatment with crude EP from *C. canadensis* 28, even higher than that of purified PGA for all tested concentrations. Here, in this study, similar high viability was observed for human dermal fibroblast cell lines (PCS-201-012) used for investigation of crude EP, and purified PGA and EP doses showed higher viability than PGA for the doses of 100 and 500 µg/mL. According to data obtained from two different cell lines from epidermis and dermis sections of human skin, it can be concluded that EP from *C. canadensis* 28 with its EP and PGA content has valuable properties for cosmeceutical exploration. 

## 5. Conclusions

In conclusion, we described a highly efficient strategy for the improvement of EP production by a halophilic bacterium *C. canadensis* 28. Its advantages suggest a good possibility for large scale production in continuous cultures. Compared to batch, the continuous cultivation reached significantly higher values of EP synthesis and productivity.

The observed high cell viability and no cytotoxicity on human dermal fibroblast cells after treating with this EP determined its potential for application in the cosmetic industry.

## Figures and Tables

**Table 1:** Process parameters in batch and continuous cultures for exopolymer (EP) production by *C. canadensis* 28.

Cultivation Condition	Dilution Rate (D)	Fluid Rate (mL/h)	Biomass, X (mg/mL)	Lactose, S (mg/mL)	EP (mg/mL)	Y_EP/X_ (mg/g)	Y_EP/S_ (mg/g)	Productivity (µg/mL/h)
Batch	-		2.9	7.2	2.34	806	0.34	48
Continuous	0.02	48	2.5	6.4	1.62	647	0.25	32.34
	0.035	84	2.5	6.6	2.15	860	0.33	75.25
	0.05	120	2.5	6.1	1.58	633	0.24	79.15
	0.075	180	2.2	5.3	1.39	631	0.20	104.17
	0.1	240	0.5	3.5	0.27	550	0.04	27.5

**Table 2:** EP content in ethanol pellet after batch and continuous cultivation.

Type of Cultivation	Dry Weight (μg/mL)	EP (μg/mL)	EP Content (%)
Batch (48 h)	3.00	2.34	78
Continuous, D = 0.02	1.84	1.62	88
Continuous, D = 0.035	2.31	2.15	93
Continuous, D = 0.05	1.66	1.58	95
Continuous, D = 0.075	1.45	1.39	96
Continuous, D = 0.1	0.29	0.27	94
